# Gastritis and AIDS-related cholangiopathy as an unusual presentation of HIV infection

**DOI:** 10.1093/gastro/goag017

**Published:** 2026-03-03

**Authors:** Fabio Brivio, Alice Covizzi, Davide Bernasconi, Guido Gubertini, Monica Schiavini, Silvia Grosso, Luca Carsana, Massimo Tonolini, Manuela Nebuloni, Andrea Gori, Emanuele Palomba

**Affiliations:** Department of Infectious Diseases, Luigi Sacco Hospital, Milan, Italy; Department of Infectious Diseases, Luigi Sacco Hospital, Milan, Italy; Department of Infectious Diseases, Luigi Sacco Hospital, Milan, Italy; Department of Infectious Diseases, Luigi Sacco Hospital, Milan, Italy; Department of Infectious Diseases, Luigi Sacco Hospital, Milan, Italy; Microbiology Unit, Luigi Sacco Hospital, Milan, Italy; Pathology Unit, Luigi Sacco Hospital, Milan, Italy; Radiology Unit, Luigi Sacco Hospital, Milan, Italy; Pathology Unit, Luigi Sacco Hospital, Milan, Italy; Department of Biomedical and Clinical Sciences ‘L. Sacco’, University of Milan, Milan, Italy; Department of Infectious Diseases, Luigi Sacco Hospital, Milan, Italy; Department of Biomedical and Clinical Sciences ‘L. Sacco’, University of Milan, Milan, Italy; Centre for Multidisciplinary Research in Health Science (MACH), University of Milano, Milan, Italy; Department of Infectious Diseases, Luigi Sacco Hospital, Milan, Italy; Centre for Multidisciplinary Research in Health Science (MACH), University of Milano, Milan, Italy

## Introduction

Acquired immune deficiency syndrome (AIDS) cholangiopathy has been a recognized clinical entity since the 1980s [1]. While the direct effects of human immunodeficiency virus (HIV) on hepatocytes and Kupffer cells are well described, the precise mechanisms leading to cholangiopathy remain incompletely understood. Current evidence suggests that, beyond HIV itself, coinfection with opportunistic pathogens plays a central role, as they trigger inflammatory responses that promote biliary strictures, obstruction, and cholestatic liver injury [2]. The condition is typically observed in individuals with advanced immunodeficiency (commonly defined as having a CD4 lymphocytes count of <0.2 × 10^9^/L). Clinically, patients often present with cholestatic liver disease and biliary dilatation, though these findings are non-specific. As a result, AIDS-related cholangiopathy remains a diagnosis of exclusion [2, 3].

Recent data regarding the global epidemiological burden of AIDS-related cholangiopathy are limited. However, several epidemiological studies have assessed cryptosporidiosis among people living with HIV (PLWH), showing that it is a rare condition, particularly in high-income countries. In these settings, the estimated incidence is less than one case per 1,000 person-years [4]. Although previous cases of cryptosporidiosis in Italian patients with clinical and radiological findings consistent with AIDS-related cholangiopathy have been reported in the literature, these date back more than a decade [5].

We describe the case of a patient who presented with gastrointestinal symptoms and was ultimately found to have AIDS-related cholangiopathy. The atypical clinical course resulted in a prolonged misdiagnosis, underlining the importance of considering this entity even in patients without an established diagnosis of HIV infection.

## Case report

A 37-year-old male presented to the Emergency Department with a 17-kg weight loss over several months, accompanied by nausea and hematemesis. He also reported intermittent low-grade fever, fatigue, and night sweats. His history included a 4-month episode of diarrhoea the previous year, during which colonoscopy showed sigmoid colitis attributed to an undetermined infection. He later developed proctalgia and rectal bleeding, leading to a diagnosis of proctitis and anal condylomas. The patient denied high-risk sexual behaviours. Inflammatory bowel disease was suspected and mesalazine was initiated, with clinical improvement.

**Figure 1:**
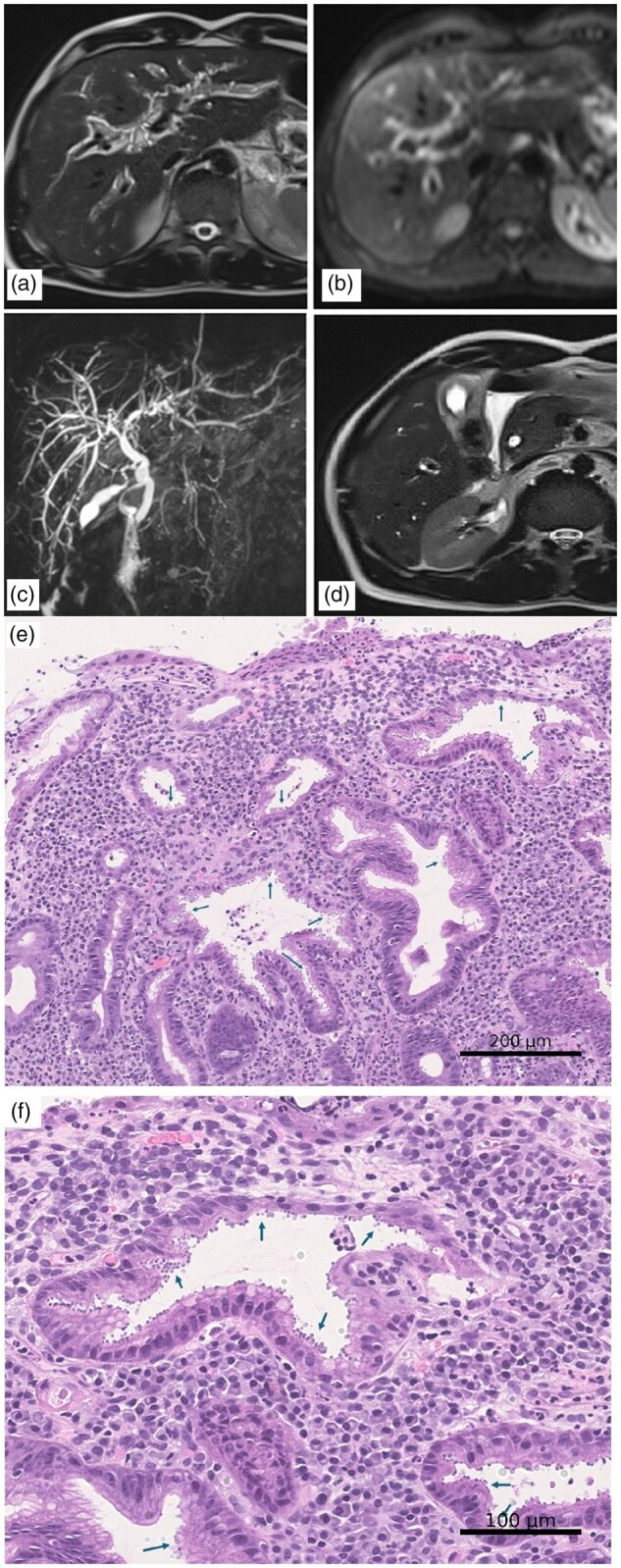
Radiological and histological findings. (a) T2-weighted images revealing a pronounced ‘periportal tracking’ in both liver lobes, with (b) corresponding hyperintense oedematous areas on diffusion-weighted imaging; (c) magnetic resonance cholangiopancreatography showing a main bile duct with a diameter of 6–7 mm, minimal intrahepatic cholangitic irregularities in the left hepatic lobe and (d) a contracted gallbladder suggestive of biliary hypofunction, with associated wall oedema. The histological image shows a portion of the superficial gastric mucosa, characterized by active chronic inflammation associated with the presence of numerous basophilic spherical bodies measuring 2–5 microns (arrows), protruding on the apical surface of the gastric epithelial cells, both superficially and at the neck regions. Haematoxylin–eosin stain, original magnifications (e) 20× and (f) 40×.

At admission, physical examination revealed mild right hypochondrial tenderness. Laboratory tests showed elevated liver enzymes (AST 176 U/L, ALT 179 U/L), leukopenia (2.6 × 10^9^/L), and normal C-reactive protein levels. The evolution of key biochemical markers is detailed in Supplementary Table S1. A contrast-enhanced abdominal computed tomography reported distended gallbladder with wall thickening, small pericholecystic fluid collection, and the dilation of intra- and extrahepatic bile ducts. Subsequently, a magnetic resonance cholangiopancreatography demonstrated diffuse periportal oedema, contracted gallbladder with wall thickening, dilated common bile duct without stones, and moderate intrahepatic ductal dilation (Fig. 1a–d). Serological tests for hepatitis B and C viruses were negative. Given the leukopenia, an HIV antigen–antibody test was performed, leading to an HIV diagnosis (CD4^+^ 0.058 × 10^9^/L, HIV-RNA 701,000 copies/mL).

The patient continued to experience nausea and vomiting, and the liver enzymes and cholestatic markers further increased (AST/ALT up to 523/510 U/L, ALP/GGT 475/618 U/L). Endoscopic ultrasound was performed, demonstrating extrahepatic bile duct dilation without stones and papillary hypertrophy. AIDS-related cholangiopathy was suspected, and stool microscopy was performed and confirmed the presence of *Cryptosporidium parvum* oocysts. Upper endoscopy with biopsy showed gastric cryptosporidiosis involving the antrum, angle, and body, associated with marked acute and chronic granulocytic inflammation (Fig. 1e and f).

Antiretroviral therapy with bictegravir/emtricitabine/tenofovir alafenamide was then initiated. The patient improved, with the resolution of fatigue, nausea, and vomiting, and liver function tests progressively normalized (Supplementary Table S1). Given this favourable response, no antiparasitic therapy was prescribed. At the 13-month follow-up on antiretroviral therapy, the patient remained asymptomatic, with normal liver enzymes and no sonographic abnormalities on abdominal ultrasound.

## Discussion

The introduction of highly active antiretroviral therapy (HAART) has transformed the epidemiological landscape of AIDS-related cholangiopathy, leading to a marked reduction in its incidence [6]. Nevertheless, the condition persists in patients with uncontrolled or undiagnosed HIV infection, particularly in those with profound immunodeficiency.

AIDS-related cholangiopathy is considered a secondary sclerosing cholangitis of infectious origin. Opportunistic pathogens act in synergy with HIV to damage the biliary tree. *Cryptosporidium parvum* is implicated in up to 57% of cases, while cytomegalovirus accounts for 10%–20% and other pathogens, such as *Microsporidia*, *Mycobacterium avium*, *Cyclospora*, and *Giardia*, are retrieved less frequently [2].

A notable prevalence of *C. parvum* is still documented among PLWH [7]. These findings underline the ongoing burden of cryptosporidial infection in PLWH, although this condition does not always cause cholangiopathy. Moreover, recent epidemiological data on cryptosporidiosis derive from low-income countries, in which risk factors and healthcare access differ from those in high-income settings.

In developed countries, AIDS-related cholangiopathy is rare and primarily affects individuals, predominantly young men who have sex with men, who are noncompliant with HAART or lack access to therapy.

The clinical spectrum of AIDS-related cholangiopathy is broad, ranging from asymptomatic forms to severe abdominal pain. Common features include nausea, vomiting, diarrhoea, weight loss, and right upper-quadrant tenderness, while pruritus and jaundice are less typical. Laboratory findings usually demonstrate a cholestatic pattern with elevated ALP and GGT, though up to one-quarter of patients may present with normal values [2]. Imaging can reveal biliary dilatation and papillary stenosis, but no pathognomonic signs exist and the overlap with other hepatobiliary conditions often delays recognition.

The clinical manifestations and laboratory abnormalities described in this case largely overlap those reported in other cases of AIDS-related cholangiopathy: a young male patient with a history of diarrhoea and weight loss, associated with altered cytolytic and cholestatic liver enzymes, in the absence of overt jaundice [8]. The absence of characteristic right upper-quadrant abdominal pain, together with severe, refractory nausea and vomiting, indicated gastrointestinal involvement extending beyond the biliary tract to include the stomach, consistently with a higher and atypical anatomical localization. Crucially, this case is distinguished by the timing and context of the cholangiopathy, which manifested as the initial presentation of a previously undiagnosed HIV infection, rather than as a complication occurring later in the course of known HIV infection in a patient who was non-adherent to HAART.

Therapeutic options remain limited. Current management relies on immune restoration through HAART, with antiparasitic agents, such as nitazoxanide or paromomycin, reserved for selected cases [9]. Endoscopic interventions may provide symptomatic relief in patients with papillary stenosis. In our patient, the prompt clinical and biochemical improvement after HAART initiation obviated the need for additional treatment. Given that the patient was HAART-naive and did not present with severe multisystem organ involvement, despite the extent of the gastric disease, it is plausible that immune reconstitution following the initiation of HAART alone was sufficient to control the parasitic infection in all involved sites [10].

In conclusion, although the incidence of AIDS-related cholangiopathy declined drastically in the HAART era, this condition remains relevant and may represent the first manifestation of HIV infection. Its non-specific clinical and laboratory features often overlap with those of other gastrointestinal and hepatobiliary diseases, contributing to delayed diagnosis. This case highlights the need for clinicians to maintain awareness of AIDS-related cholangiopathy and to consider it within the differential diagnosis when evaluating patients with advanced cholestatic liver disease of unclear origin.

## Supplementary Material

goag017_Supplementary_Data
