# Supplementary material for: Gastritis and AIDS-related cholangiopathy as an unusual presentation of HIV infection
Source: Gastroenterol Rep (Oxf). 2026 Mar 3;14:goag017. doi: 10.1093/gastro/goag017 (PMC12955836; doi:10.1093/gastro/goag017)
Supplement: goag017_Supplementary_Data [file goag017_supplementary_data.docx]

**SUPPLEMENTARY MATERIALS**

**Gastritis and AIDS-related cholangiopathy as an unusual presentation of HIV infection**

Fabio Brivio^1,†^, Alice Covizzi^1, †,*^, Davide Bernasconi^1^, Guido Gubertini^1^, Monica Schiavini^1^, Silvia Grosso^2^, Luca Carsana^3^, Massimo Tonolini^4^, Manuela Nebuloni^3,5^, Andrea Gori^1,5,6^, Emanuele Palomba^1,6^

^1^Department of Infectious Diseases, Luigi Sacco Hospital, Milan, Italy

^2^Microbiology Unit, Luigi Sacco Hospital, Milan, Italy

^3^Pathology Unit, Luigi Sacco Hospital, Milan, Italy

^4^Radiology Unit, Luigi Sacco Hospital, Milan, Italy

^5^Department of Biomedical and Clinical Sciences “L. Sacco”, University of Milan, Milan, Italy

^6^Centre for Multidisciplinary Research in Health Science (MACH), University of Milano, Milan, Italy

†Fabio Brivio and Alice Covizzi contributed equally to this paper.

***Corresponding author**.

Department of Infectious Diseases, Luigi Sacco Hospital. Via G.B. Grassi, 74 – 20157 Milan, Italy. Tel 0039 0239042545 Email: [covizzi.alice@asst-fbf-sacco.it](mailto:covizzi.alice@asst-fbf-sacco.it)

| Biomarkers  *(reference limit)* | ADMISSION^*^ | DISCHARGE | FOLLOW-UP  (13 months after admission) |
| --- | --- | --- | --- |
| HIV-RNA  *(<20 cp/ml)* | 701000 | N/A | 55 |
| CD4  *(500-1800 cells/uL)* | 58 | N/A | 281 |
| Bilirubin  *(<1.2 mg/dL)* | 0.34 | <1.2 | 0.44 |
| AST  *(<34 U/L)* | 208 | 45 | 31 |
| ALT  *(<49 U/L)* | 233 | 53 | 30 |
| GGT  *(< 68 U/L)* | 501 | 264 | 128 |
| ALP  *(<115 U/L)* | 528 | 361 | 121 |
| CRP  *(<10 mg/L)* | 1.6 | 1.3 | N/A |

**Table 1.** Trends of laboratory parameters at three different time points during the course of infection

^*^ Admission to the Infectious Diseases Unit

Legend: AST: Aspartate Aminotransferase; ALT: Alanine Aminotransferase; GGT: Gamma-Glutamyl Transferase; ALP: Alkaline Phosphatase; CRP: C Reactive Protein; N/A: not available.
